# A Comparative Study of the Efficacy and Safety of Oral Misoprostol, Intravenous Oxytocin, and Intravaginal Dinoprostone for Labor Induction in Pakistani Women

**DOI:** 10.7759/cureus.39768

**Published:** 2023-05-31

**Authors:** Asad Ullah Wasim, Muhammad Muneeb Khan, FNU Aneela, Haris Khan, Mirna Denise Díaz Solís, Insha Shabir, Syed Saem Ul Hassan, Umer Bin Tariq

**Affiliations:** 1 Medicine, Fazaia Medical College, Islamabad, PAK; 2 Clinical and Translational Research, Larkin Community Hospital, South Miami, USA; 3 Medicine and Surgery, Ayub Medical College, Abbottabad, PAK; 4 Medicine and Surgery, Liaquat University of Medical and Health Science, Jamshoro, PAK; 5 Medicine and Surgery, Jinnah Medical College, Peshawar, PAK; 6 Obstetrics and Gynaecology, UMAE Obstetrics and Gynecology Hospital, Mexico City, MEX; 7 Medicine and Surgery, Fatima Jinnah Medical University, Lahore, PAK; 8 Medicine, Nawaz Sharif Medical College, Gujrat, PAK

**Keywords:** dinoprostone, oxytocin, maternal and perinatal outcome, misoprostol, obstetric outcome, labor induction

## Abstract

Introduction: A frequent medical procedure to accelerate labor is the induction of labor. There are different methods of labor induction, including the use of medications such as misoprostol, oxytocin, and dinoprostone.

Objective: This research compared the effectiveness and safety of oral misoprostol, intravenous oxytocin, and intravaginal dinoprostone for labor induction in Pakistani women.

Methodology: A study was conducted in the Department of Obstetrics and Gynaecology, Hayatabad Medical Complex-Medical Teaching Institute (MTI) and Lady Reading Hospital-MTI, Peshawar, Pakistan, over two years. It included 378 women between 38 and 42 gestational weeks, divided into three groups of 126 women each. The oral misoprostol group was given a maximum of six doses of a 25 μg oral misoprostol solution (oral misoprostol tablet of 200 μg dissolved in 200 ml) at intervals of two hours. The drip rate for the intravenous oxytocin group ranged from 6 mIU/minute to 37 mIU/minute. The intravaginal dinoprostone group received a controlled-release vaginal insert containing 10mg of intravaginal dinoprostone, which was left in place for 12 hours.

Results: More women in the oral misoprostol group (n=94; 74.6%) had successful inductions when compared to the intravaginal dinoprostone (n=83; 65.9%) and intravenous oxytocin (n = 77; 64.71%) groups. Oral misoprostol had the greatest proportion of normal vaginal deliveries (n=62; 65.95%), followed by intravaginal dinoprostone (n=47; 56.63%), and intravenous oxytocin had the lowest rate (n=33; 42.85%). Cesarean section rates were greatest in the intravenous oxytocin group (n=31; 40.26%), followed by the intravaginal dinoprostone group (n=29; 34.94%), and lowest in the oral misoprostol group (n=24; 25.53%).

Conclusion: Oral misoprostol induces labor in women safely and effectively, resulting in the lowest percentage of cesarean deliveries and the highest percentage of normal vaginal deliveries, respectively. Intravaginal dinoprostone showed the lowest rate of side effects, followed by oral misoprostol while intravenous oxytocin had the highest rate of side effects.

## Introduction

Induction of labor is a medical intervention that is used to start and speed up labor when it does not begin naturally or progress as expected. This procedure can be performed for various reasons, such as maternal or fetal health concerns, post-term pregnancy, or failed membrane rupture. There are several methods for inducing labor, including membrane sweeping or stripping, Foley bulb catheter, mechanical or manual dilation, rupturing the amniotic membrane, and medications i.e., the use of oxytocin hormone and prostaglandins such as misoprostol and dinoprostone, etc. Induction of labor is a carefully monitored process by healthcare providers to ensure the safety of both the mother and the baby [1-3].

Misoprostol, oxytocin, and dinoprostone are three medications commonly used for labor induction. Misoprostol is a synthetic prostaglandin E1 analogue that can be administered orally to stimulate the cervix to soften and dilate, as well as stimulate uterine contractions. It has been shown to be effective in inducing labor and is often used when cervical ripening is needed. However, misoprostol has been associated with a higher incidence of uterine hyperstimulation, which can lead to fetal distress, uterine rupture, and other complications [4-6].

Oxytocin is a naturally occurring hormone that induces uterine contractions and is commonly used for the induction of labor. It is administered through an intravenous infusion and is associated with fewer cases of uterine hyperstimulation compared to misoprostol. However, oxytocin use can also result in adverse outcomes, such as fetal distress, uterine rupture, and cesarean delivery [7-9].

Dinoprostone, a prostaglandin E2 analogue, is another medication used for labor induction. It is available in various forms, including intravaginal tablets, gels, and sustained-release vaginal inserts. Like misoprostol, it can cause uterine hyperstimulation and requires close monitoring [10,11].

No direct comparison of the three commonly used medications (oral misoprostol, intravenous oxytocin, and intravaginal dinoprostone) for labor induction or cervical ripening exists in the current literature. Thus, the research objective was to compare the effectiveness and safety of oral misoprostol, intravenous oxytocin, and intravaginal dinoprostone for labor induction in Pakistani women. The comparison of these medications is crucial to optimizing patient care and developing new protocols for their use. By exploring the relative merits and limitations of each medication, healthcare providers will be better equipped to make informed decisions about which medication to prescribe in different clinical scenarios. Furthermore, this research could contribute to the development of new guidelines or protocols for the administration of these medications, potentially leading to improved clinical outcomes.

## Materials and methods

Study design

The study was conducted in the Department of Obstetrics and Gynaecology, Hayatabad Medical Complex-Medical Teaching Institute (MTI), and Lady Reading Hospital-MTI in Peshawar, Pakistan, from January 1, 2021 to 31 Decemeber, 2022. This study was approved by the research ethics committees of both hospitals (approval no. 2318). Each patient provided written consent ahead of their participation in the trial.

Inclusion and exclusion criteria

The following inclusion criteria were applied in this study: women between the ages of 18 and 40, a Bishop's score of six or below, cephalic presentation, healthy fetal heart rate, and singleton pregnancy. Women who were unwilling to participate in the study or had an antepartum hemorrhage or cephalopelvic disproportion were excluded from the study. Also excluded were women in the oral misoprostol and intravaginal dinoprostone groups who entered active labor after 24 hours, as well as those in the intravenous oxytocin group who did so after 12 hours. Women with fetal distress or non-reassuring fetal heart rate patterns, known or suspected placenta previa or placental abruption, and a history of uterine surgery or major uterine anomaly, were also excluded from the study.

Sample size

During the trial, 378 of the 672 women (between 38 and 42 gestational weeks) who were hospitalized for induction satisfied the requirements and underwent evaluation. They were split into three groups of 126 women each and given either oral misoprostol, intravenous oxytocin, or intravaginal dinoprostone for labor induction.

Sampling technique

The oral misoprostol group was given a maximum of six doses of a 25 μg oral misoprostol solution (oral misoprostol tablet of 200 μg dissolved in 200 ml) at intervals of two hours. Six doses were administered, and if contractions failed to begin within 12 hours, no more doses were given, and the patients were monitored for another 12 hours. The women in the intravenous oxytocin group received titrating doses of 5 IU of oxytocin in 500 mL of Ringer's lactate at rates ranging from 10 drops per minute (6 mIU/minute) to 60 drops per minute (37 mIU/minute). The women in the intravaginal dinoprostone group received a controlled-release vaginal insert containing 10 mg of dinoprostone, which was left in place for 12 hours.

Effective induction was considered to have taken place when women entered the active phase of labor within the allotted time frames: 12 hours for those who received intravenous oxytocin and 24 hours for participants who received oral misoprostol or intravaginal dinoprostone.

Data analysis

The statistical analysis was performed using Statistical Product and Service Solutions (SPSS) software version 23 (IBM Corp., Armonk, NY, USA). Various parameters including the women's age, the period between labor onset and active phase, Bishop's score, and the duration between labor and delivery, were reported as means as well as standard deviations. An independent sample t-test was used to compare the average values of numerical numbers. A chi-square test was employed to compare the differences in percentages of categorical data such as gestational week, parity, body mass index, the period between labor onset and its active phase, the duration between labor and delivery, methods of delivery, and maternal complications. Statistical significance was defined as a p-value of 0.05.

## Results

This study examined 378 women who underwent labor induction. The initial demographic characteristics (such as maternal age, gestation period, and maternal parity) and Bishop's score did not show any significant differences among the three groups (Table 1).

**Table 1:** Analysis of demographic details and Bishop's score in participants

Category		Oral misoprostol (n=126)	Intravenous oxytocin (n=126)	Intravaginal dinoprostone (n=126)	p-value
Maternal age (years)	Less than 20	5 (3.96%)	7 (5.55%)	9 (7.14%)	0.49
	20-25	48 (38.09%)	37 (29.36%)	43 (34.12%)	
	26-30	57 (45.24%)	59 (46.83%)	49 (38.89%)	
	31-35	13 (10.32%)	17 (13.49%)	21 (16.67%)	
	Greater than 35	3 (2.39%)	6 (4.77%)	4 (3.18%)	
	Mean age ± SD (years)	24.39 ± 3.69	25.02 ± 3.21	23.63 ± 2.37	0.843
Period of gestation (weeks)	38	14 (11.11%)	23 (18.25%)	18 (14.28%)	0.17
	39	23 (18.25%)	27 (21.43%)	29 (23.01%)	
	40	77 (61.11%)	63 (50.0%)	68 (53.97%)	
	41	9 (7.14%)	11 (8.73%)	7 (5.56%)	
	42	3 (2.39%)	2 (1.59%)	4 (3.18%)	
Maternal parity	0	79 (62.69%)	82 (65.08%)	75 (59.52%)	0.273
	1	42 (33.35%)	34 (26.98%)	45 (35.71%)	
	2	5 (3.96%)	8 (6.35%)	4 (3.18%)	
	3	0 (%)	2 (1.59%)	2 (1.59%)	
Bishop's score	Mean (SD)	3.86 (0.68)	3.81 (0.63)	3.77 (0.61)	0.568

The rate of successful induction among the three groups were compared (Table 2). A higher proportion of women (n=94; 74.6%) in the oral misoprostol group experienced effective inductions, followed by the intravaginal dinoprostone (n=83; 65.9%) and intravenous oxytocin groups (n=77; 64.71%).

**Table 2:** Successful induction of study subjects

Parameters	Oral misoprostol group (n=126)	Intravenous oxytocin group (n=126)	Intravaginal dinoprostone (n=126)	p-value
Failed induction	32 (25.4%)	49 (35.29%)	43 (34.1%)	0.358
Successful induction	94 (74.6%)	77 (64.71%)	83 (65.9%)	

The duration of induction to active labor in hours for the three groups, i.e., oral misoprostol, intravenous oxytocin, and intravaginal dinoprostone, is shown in Table 3. The numbers in each column represent the percentage of participants in each group who gave birth within the corresponding timeframe. The mean onset of active labor was 9.51 ± 4.62 hours in the oral misoprostol group, 6.19 ± 3.21 hours in the intravaginal dinoprostone group, and 4.69 ± 2.36 hours in the intravenous oxytocin group. Hence, the duration of induction to active labor was dramatically reduced in the intravenous oxytocin group (Table 3).

**Table 3:** Start of the active labor period in study participants

Start of active labor period (hours)		Oral misoprostol (n=94)		Intravenous oxytocin (n=77)		Intravaginal dinoprostone (n=83)	
Less than 6 hours		23 (24.5%)		31 (40.25%)		19 (22.89%)	
6 to 12 hours		32 (34.0%)		46 (59.75%)		37 (44.58%)	
Greater than 12 hours and less than 24 hours		39 (41.5%)		0 (0)		27 (32.53%)	
Total		94		77		83	
Mean onset time of active labor (hours)							
Oral misoprostol		Intravenous oxytocin		Intravaginal dinoprostone		t-test	p-value
Mean	Standard deviation	Mean	Standard deviation	Mean	Standard deviation		
9.51	4.62	4.69	2.36	6.19	3.21	5.49	<0.002

Women who gave birth vaginally on average took 11.35 hours on oral misoprostol, 8.89 hours with intravaginal dinoprostone, and 6.57 hours with intravenous oxytocin to complete their deliveries (Table 4).

**Table 4:** Comparison of vaginal births from initiation of induction to time of delivery

Initiation of induction to time of delivery (hours)		Oral misoprostol (n=94)		Intravenous oxytocin (n=77)		Intravaginal dinoprostone (n=83)	
Less than 6 hours		16 (17.02)		24 (31.17)		12 (14.46)	
6 to 12 hours		29 (30.85)		39 (50.65)		21 (25.30)	
Greater than 12 hours and less than 24 hours		46 (48.94)		14 (18.18)		41 (49.40)	
Greater than 24 hours		3 (3.19)		0		9 (10.84)	
Mean delivery time (hours)							
Oral misoprostol		Intravenous oxytocin		Intravaginal dinoprostone		t-test	p-value
Mean	Standard deviation	Mean	Standard deviation	Mean	Standard deviation		
11.35	6.33	6.57	2.90	8.89	5.32	5.49	<0.002

The majority of women in all three groups had a normal vaginal delivery, with the highest percentage observed in the oral misoprostol group (n=62; 65.95%) and the lowest in the intravenous oxytocin group (n=33; 42.86%). The rates of cesarean section were highest in the intravenous oxytocin group (n=31; 40.26%) and lowest in the oral misoprostol group (n=24; 25.53%). The rates of tachysystole or hyperstimulation, which is an excessive contraction of the uterus, were highest in the intravenous oxytocin group (n=11; 14.28%) and lowest in the intravaginal dinoprostone group (n=2; 2.41%) (Table 5).

**Table 5:** Induction results and maternal complications

Delivery method	Oral misoprostol (n=94)	Intravenous oxytocin (n=77)	Intravaginal dinoprostone (n=83)	p-value
Normal vaginal delivery	62 (65.95%)	33 (42.86%)	47 (56.63%)	0.162
Instrumental vaginal delivery	4 (4.26%)	2 (2.60%)	5 (6.02%)	
Cesarean	24 (25.53%)	31 (40.26%)	29 (34.94%)	
Tachysystole/Hyperstimulation	4 (4.26%)	11 (14.28%)	2 (2.41%)	0.029

## Discussion

Labor induction is a commonly used intervention in obstetric care, and several medications are used for this purpose, including but not limited to misoprostol, oxytocin, and dinoprostone. These medications work through different mechanisms to initiate and promote uterine contractions, leading to the onset of labor. This comparative study aimed to evaluate the efficacy and safety of these three medications for labor induction in Pakistani women.

Our research revealed a higher occurrence of normal vaginal deliveries among patients who received oral misoprostol compared to those who received intravaginal dinoprostone and intravenous oxytocin. Aalami-Harandi et al. conducted a study using 12 doses of 25 μg of oral misoprostol and revealed that patients getting oral misoprostol had a noticeably higher percentage of vaginal births at 18 and 24-hour intervals during the study period, in comparison to patients receiving intravenous oxytocin [12]. However, in a study conducted by Faucett et al., there was no significant difference in vaginal delivery rates between women who received intravaginal dinoprostone and those who received oral misoprostol [13].

In our study, we noted that patients who received intravenous oxytocin experienced a significantly shorter duration of labor induction (4.69 ± 2.36) until reaching active labor, in comparison to those who received oral misoprostol and intravaginal dinoprostone (9.51 ± 4.62 and 6.19 ± 3.21, respectively). When examining maternal complications, we observed a higher occurrence of hyperstimulation in patients receiving intravenous oxytocin (14.28%) as opposed to intravaginal dinoprostone (2.41%) and oral misoprostol (4.26%). Aalami-Harandi et al. conducted a similar study and arrived at similar conclusions, highlighting that patients receiving intravenous oxytocin had shorter durations until active labor and a higher incidence of hyperstimulation compared to patients receiving oral misoprostol [12]. A study by Escudero et al. found a higher incidence of hyperstimulation among both oral misoprostol and intravenous oxytocin [14]. In a study by Nunes et al., a low incidence of hyperstimulation was seen in patients receiving intravaginal dinoprostone, which is consistent with our results [15].

In our study, the highest percentage of normal vaginal deliveries was in the oral misoprostol group at 65.95%, followed by the intravaginal dinoprostone group at 56.63%, and then the intravenous oxytocin group at 42.86%, which showed that oral misoprostol is most effective. Comparable outcomes were observed by Aalami-Harandi et al., who found an increased rate of normal vaginal delivery in the oral misoprostol group compared to the intravenous oxytocin group [12]. Similarly, Kulshreshtha et al. found more normal vaginal deliveries induced in the oral misoprostol group compared to the intravaginal dinoprostone group [16]. Further, the highest percentage of cesarean deliveries was in the intravenous oxytocin group at 40.26%, followed by the intravaginal dinoprostone group at 34.94%, and then the oral misoprostol group at 25.53%, which showed that oral misoprostol is safer for labor induction. Similar findings were observed by Das et al., who found more cesarean deliveries in the intravenous oxytocin group and intravaginal dinoprostone group [17,18].

## Conclusions

This comparative study suggests that oral misoprostol may be more effective for labor induction in Pakistani women as it resulted in higher rates of normal vaginal deliveries compared to intravenous oxytocin and intravaginal dinoprostone. However, intravenous oxytocin was found to be more efficient in initiating active labor and delivering the baby in a shorter timeframe. Maternal complications such as hyperstimulation were observed more frequently in patients receiving intravenous oxytocin compared to oral misoprostol and intravaginal dinoprostone. Furthermore, the incidence of cesarean delivery was lowest in the oral misoprostol group, indicating that it may be a safer option for labor induction. These findings provide valuable insights for clinicians to make informed decisions while choosing a suitable medication for labor induction in Pakistani women.
